# Genome-wide identification and expression profile analysis of trihelix transcription factor family genes in response to abiotic stress in sorghum [*Sorghum bicolor* (L.) Moench]

**DOI:** 10.1186/s12864-021-08000-7

**Published:** 2021-10-14

**Authors:** Kuiyin Li, Lili Duan, Yubo Zhang, Miaoxiao Shi, Songshu Chen, Mingfang Yang, Yanqing Ding, Yashu Peng, Yabing Dong, Hao Yang, Zhenhua Li, Liyi Zhang, Yu Fan, Mingjian Ren

**Affiliations:** 1 College of Agriculture, Guizhou University, Guiyang, 550025 People’s Republic of China; 2 College of Agriculture, Anshun University, Anshun, 561000 People’s Republic of China; 3 Institute of Upland Food Crops, Guizhou Academy of Agricultural Sciences, Guiyang, 550006 Guizhou People’s Republic of China; 4 Guizhou Branch of National Wheat Improvement Center of Guizhou University, Huaxi District, Guiyang, 550025 Guizhou Province People’s Republic of China

**Keywords:** *Sorghum bicolor* (L.), Transcription factors, *Trihelix* gene, Abiotic stress, Gene duplication, Synteny

## Abstract

**Background:**

Transcription factors, including trihelix transcription factors, play vital roles in various growth and developmental processes and in abiotic stress responses in plants. The *trihelix* gene has been systematically studied in some dicots and monocots, including *Arabidopsis,* tomato, chrysanthemum, soybean, wheat, corn, rice, and buckwheat. However, there are no related studies on sorghum.

**Results:**

In this study, a total of 40 sorghum trihelix (*SbTH*) genes were identified based on the sorghum genome, among which 34 were located in the nucleus, 5 in the chloroplast, 1 (*SbTH38*) in the cytoplasm, and 1 (*SbTH23*) in the extracellular membrane. Phylogenetic analysis of the *SbTH* genes and *Arabidopsis* and rice *trihelix* genes indicated that the genes were clustered into seven subfamilies: SIP1, GTγ, GT1, GT2, SH4, GTSb8, and orphan genes. The *SbTH* genes were located in nine chromosomes and none on chromosome 10. One pair of tandem duplication gene and seven pairs of segmental duplication genes were identified in the *SbTH* gene family. By qPCR, the expression of 14 SbTH members in different plant tissues and in plants exposed to six abiotic stresses at the seedling stage were quantified. Except for the leaves in which the genes were upregulated after only 2 h exposure to high temperature, the 12 *SbTH* genes were significantly upregulated in the stems of sorghum seedlings after 24 h under the other abiotic stress conditions. Among the selected genes, *SbTH10/37/39* were significantly upregulated, whereas *SbTH32* was significantly downregulated under different stress conditions.

**Conclusions:**

In this study, we identified 40 trihelix genes in sorghum and found that gene duplication was the main force driving *trihelix* gene evolution in sorghum. The findings of our study serve as a basis for further investigation of the functions of *SbTH* genes and providing candidate genes for stress-resistant sorghum breeding programmes and increasing sorghum yield.

**Supplementary Information:**

The online version contains supplementary material available at 10.1186/s12864-021-08000-7.

## Background

Abiotic stress can affect the growth process of plants considerably, reducing plant development and crop yield [1]. In view of this, plants have evolved a complex system to regulate their adaptability to stress signals [2, 3]. Transcription factors are ubiquitous in plants and play important roles in various growth and developmental processes and abiotic stress response [4]. More than 60 transcription factor families have been identified in plants [5, 6]. Nevertheless, the functions of several crucial transcription factor families have not been completely clarified. In the 1980s, the trihelix transcription factors exist only in plants and separated from the pea (*Pisum sativum*) for the first time [7]. They bind to the core sequence of 5 ‘-G-Pu- (T / A) -A- (T / A) -3 ‘of the promoter region of *rbcS-3A* gene to regulate light-dependent expression [8]. Trihelix transcription factors were initially called GT factors because they bind to photosensitive GT elements. The DNA-binding domain of GT factor has a typical helix-loop-helix-loop-helix structure, which is responsible for the name trihelix transcription factor. Studies have shown that the trihelix structure of GT factors is highly similar to the structure of Myb/SANT-LIKE DNA-binding domains [9]. GT factors evolved from Myb/SANT-LIKE proteins. The gaps between helix pairs result in different recognition sequences between GT factors and Myb/SANT-LIKE proteins [9, 10].

The *trihelix* gene has been systematically studied in some dicots and monocots, including *Arabidopsis*, tomato, chrysanthemum, soybean, wheat, corn, rice, and buckwheat. However, the trihelix family in sorghum have not been systematically studied. Because of the important functions of *trihelix* genes in tissue development, environmental adaptation and evolution, it is of great significance to systematically analyze the trihelix family members of sorghum. Presently, a total of 30 GT family members have been identified in *Arabidopsis thaliana* and were classified into GT-1, GT-2, GTγ, SH4, and SIP1 subfamilies, named after their founding members [11]. Similarly, 96 trihelix proteins have been identified in tomato and were classified into six subfamilies (GT-1, GT-2, SH4, SIP1, GTγ, and GTδ) [12]. The structures of most *trihelix* genes vary among plant species, especially at the C-terminal.

Some studies have reported the involvement of *trihelix* gene family in complex physiological functions. In *Arabidopsis*, *GT1* subfamily genes may be involved in salt stress and pathogen infection response, and their expression was induced by light in 3-days-old seedlings [13]. Additionally, expression of *RML1* of tomato *GT-1* gene was inhibited by light in yellow seedlings [14]. Osmotic, salt, and cold stress induced the expression of trihelix transcription factors, *GmGT-2A* and *GmGT-2B*, in soybeans [15]. In *Arabidopsis thaliana*, *GTL1* gene mutants can significantly reduce transpiration and improve drought tolerance [16].. The expression of GTγ evolution branch gene, *OsGTγ*-*1*, in tomato increased by 2.5 to 10 folds in response to salt stress, and the abscisic acid (ABA) treatment also upregulated its expression [17]. The *Arabidopsis SIP1* genes, *ASIL1* and *ASIL2*, downregulated the expression of *LEA* (rich in late embryogenesis) gene in *Arabidopsis* seedlings [11]. *Trihelix* genes play multiple functions during plant development. Therefore, it is necessary to clarify their roles and the molecular mechanisms involved in signal transduction pathways in different stress response.

Sorghum (*Sorghum bicolor* L. Moench) is an important food crop and is widely cultivated in different regions of the world, making it an ideal C4 plant for research. In this study, the *trihelix* gene family was identified in sorghum. The chromosomal distributions, protein characteristics, gene structures, and conserved motif compositions of the identified *trihelix* genes were analysed. We then identified orthology relations, analysed gene duplication events, and constructed phylogenetic trees of the identified *trihelix* genes. Additionally, we examined the expression pattern of selected sorghum *trihelix* genes under abiotic stresses.

## Results

### Identification of *trihelix* genes and analysis of their physicochemical properties in *S. bicolor* (L.)

The Hidden Markov Model (HMM) profile of trihelix domain (PF13837) was used to search the trihelix domain in the entire sorghum genome. Only genes with E value < 0.01 were classified as those of trihelix family. The Pfam and InterPro databases were used to confirm that the putative genes contained the Myb/SANT-LIKE domain. Finally, a total of 40 non-redundant *trihelix* genes were identified in sorghum. The sorghum *trihelix* genes were named from *Sbtrihelix1*-*Sbtrihelix40* according to their positions on the chromosome. Sbtrihelix was abbreviated to SbTH. Table S1 contains a summary of the characteristics of SbTH, including gene ID, chromosome location, coding sequence (CDS) length and amino acid sequence, protein size, and isoelectric point (PI). *SbTH5* encodes the smallest protein with 205 amino acids, whereas *SbTH18* encodes the largest protein with 875 amino acids. The SbTH protein molecular weight (Mw) ranged from 22.68 kDa–96.29 kDa, while the predicted isoelectric point ranged from 4.42 (*SbTH17*) to 11.19 (*SbTH31*). The results of subcellular localization prediction of SbTH proteins showed that 34 *SbTH* genes were located in the nucleus, 5 in the chloroplast, and 1 (*SbTH23*) in the extracellular membrane. Among the 40 *SbTH* genes, 8 (20.0%) contained the GT1 domain, 23 (57.5%) contained the Myb_DNA-binding domain and 9 (22.5%) contained both GT1 domain and Myb DNA-binding domain. The ratio of *SbTH* genes to total genes in the *S. bicolor* genome was about 0.12% [18], which is similar to that of *Arabidopsis* (0.11%) [19, 20], soybean (0.14%) [21, 22], and rice (0.10%) [23, 24] but more that than of tomato (0.05%) [12, 25], chrysanthemum (0.04%) [26, 27], wheat (0.08%) [28, 29], and buckwheat (0.06%) [30, 31].

### Phylogenetic analysis of *trihelix* genes in *S. bicolor* (L.)

**Fig. 1:**
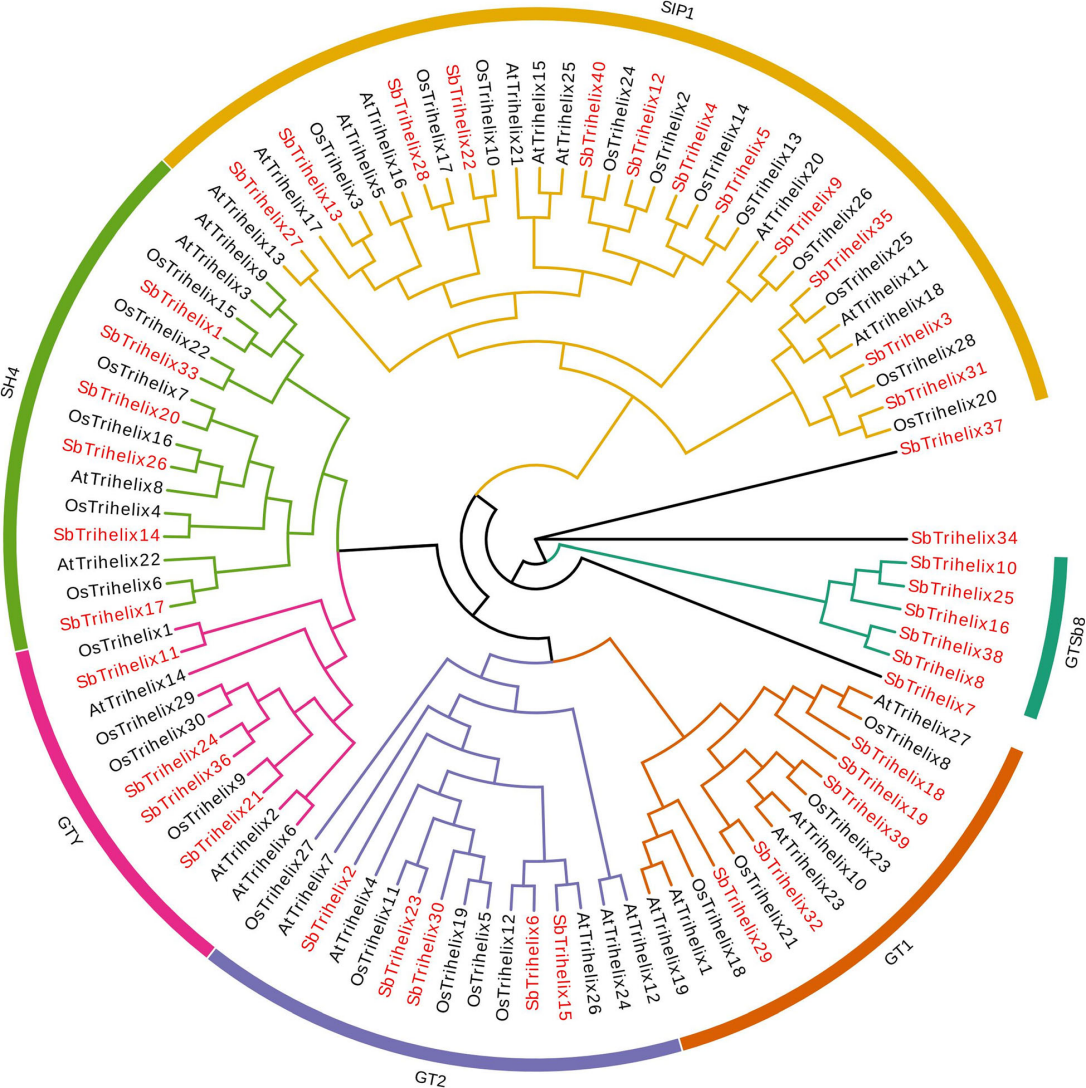
Unrooted phylogenetic tree showing relationships among Trihelix domains of *S. bicolor* and *Arabidopsis*. The phylogenetic tree was derived using the NJ method in MEGA7.0. The tree shows the 24 phylogenetic subfamilies and 1 unclassified group (*GTSb8*) marked with red font on a white background. Trihelix proteins from *Arabidopsis* are marked with the prefix ‘At’

To better understand the phylogenetic relationship of *trihelix* genes, we constructed a phylogenetic tree using the neighbour-joining (NJ) method with a bootstrap value of 1000 based on the amino acid sequences of 40 SbTH proteins, 27 *Arabidopsis thaliana* trihelix (AtTH), and 29 *Oryza sativa* trihelix (OsTH) proteins (Fig. 1, Additional file 1: Table S1). According to the topological structure of the tree and classification method proposed by Kaplan-Levy and Qin [32, 33], the 40 *trihelix* genes were clustered into six groups (SIP1, GTγ, GT1, GT2, SH4, GTSb8) and three ‘orphan genes’. Among the 40 *trihelix* genes, 32 *SbTH* genes were clustered into five subfamilies, which was consistent with the results of *Arabidopsis* and rice. Five genes (*SbTH8*, *SbTH10*, *SbTH16*, *SbTH25,* and *SbTH38*) formed an unknown significant branch. According to the classification characteristics of *trihelix* gene family, we named it GTSb8. This may represent a new evolutionary branch of the *trihelix* gene family in sorghum. Additionally, three genes (*SbTH07/34/37*) branched independently and were named ‘orphan genes’, suggesting that the three genes may have unique functions. The SIP1, GTγ, SH4, GT1, GT2, and GTSb8 subfamilies contained 12, 4, 6, 5, 5, and 5 *SbTH* genes, respectively. There were no differences in the sequences of the 32 proteins shared with *Arabidopsis thaliana* and rice during *S. bicolor* evolution, however, eight gene duplication events occurred in the sorghum genome.

### Gene structure and motif analysis of *trihelix* genes in *S. bicolor* (L.)

**Fig. 2:**
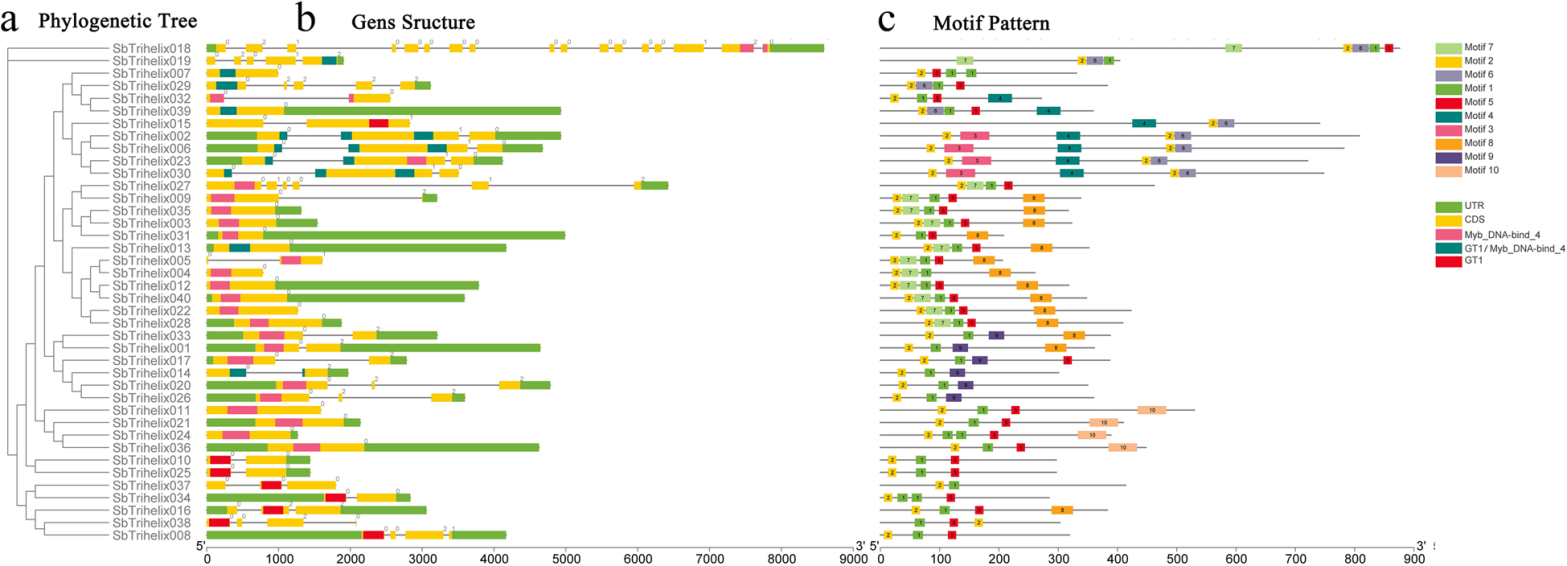
Phylogenetic relationships, gene-structure analysis, and motif distributions of *S. bicolor trihelix* genes a Phylogenetic tree was constructed using the NJ method with 1000 replicates on each node. b Exons and introns are indicated by yellow rectangles and gray lines, respectively. c Amino acid motifs in the Sbtrihelix proteins (1–10) are represented by coloured boxes. The black lines indicate relative protein lengths.

Structures and phases of introns/exons were determined by aligning the genomic DNA with full-length cDNAs of *SbTH* genes. Generally, trihelix members grouped in the same branch shared similar exons/introns organization based on the exon/intron number (Fig. 2a, b). Structural characteristics of the *SbTH* genes, including the number and distribution of exons and introns, are shown in Fig. 2b. The CDS of more than half (25,62%) of the *trihelix* genes were isolated by the introns. By analysing the gene structural characteristics, we determined that 15 (38%) of the *SbTH* genes had no intron, 11 (28%) had only 1 intron, 8 (20%) had 2 introns, 2 (5%) had 3 introns, and 2 (5%) had 4 introns, and *SbTH27* (2.5%) and *SbTH18* (2.5%) contained 5 and 16 introns, respectively. The number of exons in the SbTH family varied from 1 to 17, with the GT1 subfamily having 1–17 exons, SIP1 subfamily having 1–6, GTSb8 subfamily having 2–4 exons, three *orphan genes* having 1–3, GT2 and SH4 subfamilies having 2–3, while GTγ subfamily had only 1 exon and no intron. The GTγ subfamily contained the average lowest number of exons, whereas the GT1 family contained the highest.

As shown in Fig. 2c, we used the MEME search tool (http:/meme.nbcr.net/meme/intro.html) to predict 10 conserved motifs (motif 1 to motif 10) of SbTH proteins to further analyze the diversity of sorghum *trihelix* genes. The lengths of the conserved motifs varied from 15 to 50 amino acids. The motif organizations of each SbTH protein are shown with the corresponding colour boxes in Fig. 2c. In Additional File 2 (Table S2), the detailed sequence of each motif is provided. Motif 1 and motif 2 exist in almost all SbTH proteins, and all SbTH proteins contain motif 2. Different groups shared similar motifs, suggesting that these conserved motifs might play significant roles in particular functions. Moreover, some SbTH contained more than one motif 2. For instance, *SbTH02*, *SbTH06*, *SbTH23*, and *SbTH30*, which are members of the GT2 subfamily, contained two motifs 2. However, *SbTH15*, which is also a member of GT2 subfamily, contained no motif 3 and only one motif 2. Most members of the SIP1 subfamily contained motifs 1, 2, 5, 7 and 8, except for *SbTH27*, *SbTH31*, and *SbTH04*. Members of the SH4 subfamily contained motifs 1, 2, and 9, *SbTH33* and *SbTH01* also contained motif 8, and *SbTH17* contained motif 5. Members of the GTγ subfamily contained motifs 1, 2, 5, and 10, and *SbTH24* contained two motifs 1. There were two motifs 1 at the same time in *SbTH07* and *SbTH34*, which may be linked to their unique functions.

### Chromosome distribution and synteny analysis of *trihelix* gene in *S. bicolor* (L.)

**Fig. 3:**
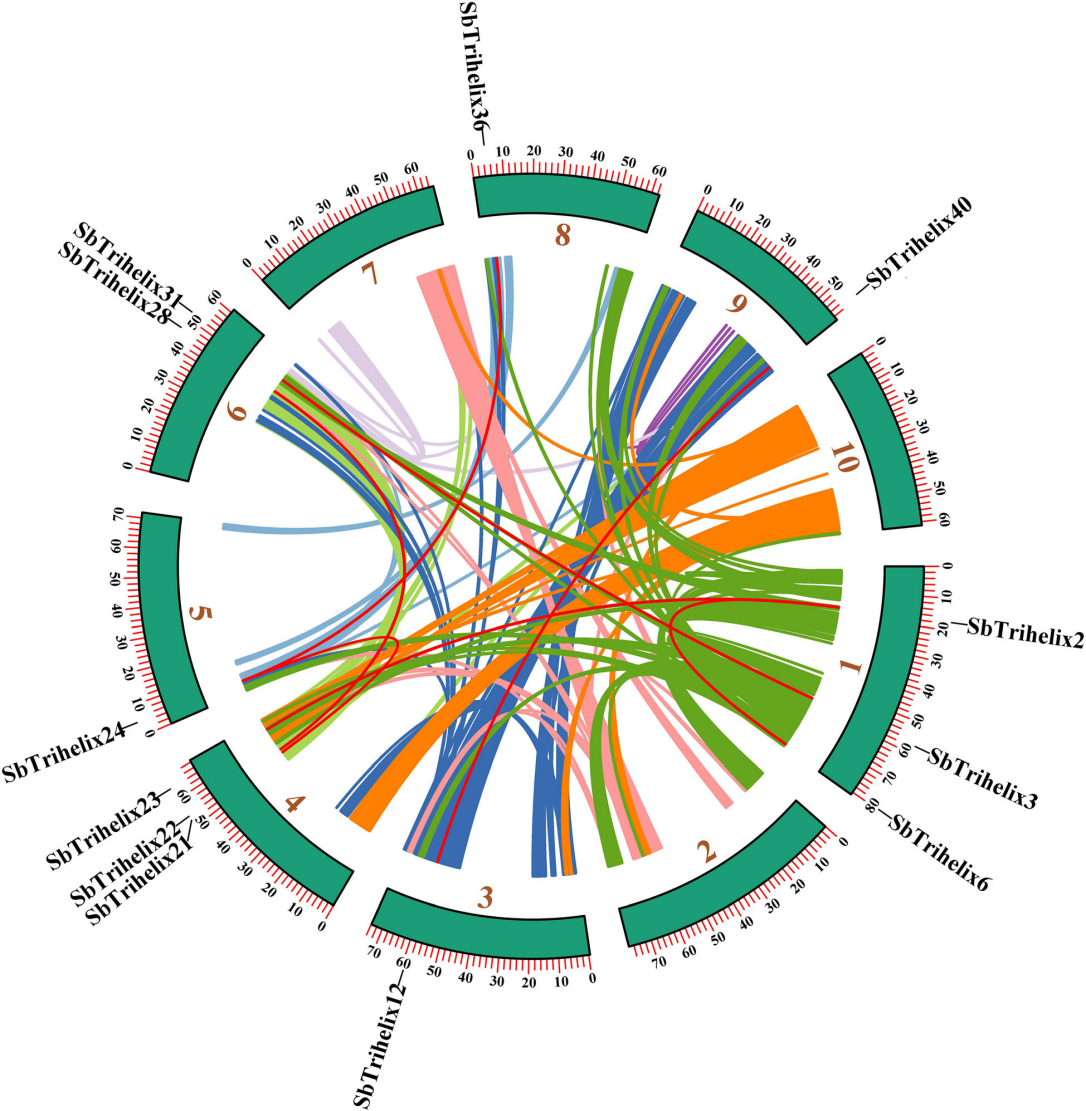
Schematic representation of the chromosomal distribution and interchromosomal relationships of *S. bicolor trihelix* genes. Coloured lines indicate all synteny blocks in the *S. bicolor* genome, and the red lines indicate duplicated *trihelix* gene pairs. Chromosome number is indicated at the bottom of each chromosome

The chromosome positions of *SbTH* genes were extracted from the genome annotation files. As shown in Fig. S1, the 40 *SbTH* genes are unevenly and non-randomly distributed at precise positions on chromosome 1 to chromosome 9. The *SbTH* genes were named according to their physical positions on the *S. bicolor* chromosome from top to bottom. Chromosome 4 (Chr4) contained the largest number of *SbTH* genes (9, 22.5%), followed by Chr6 (8, 20%), Chr1 (6, 15%), Chr2 and Chr3 (4 genes each, 10%), Chr8 (3, 7.5%), and Chr5, Chr7, and Chr9 (2 genes each, 5%). *SbTH04* and *SbTH05* formed a tandem repeat at one end of Chr1 to form a gene cluster. Except for one *SbTH* gene in the middle of Chr2, the others were unevenly and non-randomly distributed on both ends of the chromosome. Additionally, there was a pair of tandem duplication gene (*SbTH04/SbTH05*, SIP1 subfamily gene) on chr1 and seven pairs of segmental duplication genes (Fig. 3, Additional File 3: Table S3). A chromosomal region within 200 kb exhibiting two or more identical genomic regions is defined as a tandem duplication event.

As shown in Fig. 3, 13 (32.5%) paralogs were identified in the *SbTH* gene family, indicating an evolutionary relationship among these SbTH members. The *SbTH* genes were unevenly distributed in 10 *S. bicolor* linkage groups (LGs) (Fig. 3). Some LGs had more *SbTH* genes than others (LG1, LG4), with LG1 having the most *SbTH* genes (3). Further analysis of the subfamilies of these genes showed that all of them were linked within their subfamily. Among the six *SbTH* gene subfamilies, the SIP1 subfamily had the largest number of linked genes (6/13), whereas the GT2 and GTγ subfamilies had 3 linked genes each.

### Evolutionary and synteny analyses between *SbTH* genes and those of several other species

To analyse the evolutionary relationship of the *trihelix* gene family between sorghum and five plants (*Arabidopsis*, wheat, rice, tomato, and buckwheat), an unrooted NJ tree with 10 conserved motifs was constructed using the NJ method of Geneious R11 according to the protein sequences of 40 *SbTH* genes and the *trihelix* genes of five other plants (Fig. S2, the detailed genetic correspondence can be found in Additional File 4: Table S4). The distribution of SbTH in the phylogenetic tree was relatively dispersed. Most members of the trihelix family from different species, shown in Fig. S2, shared motifs 2, and most trihelix family members contained motifs 1 and motifs 5. Generally, trihelix proteins in the same subfamily had similar motif compositions, and similar serial motifs tended to cluster in sorghum, wheat, and rice, indicating that SbTH proteins may be more closely related to those of rice and wheat than those of the other plants.

**Fig. 4:**
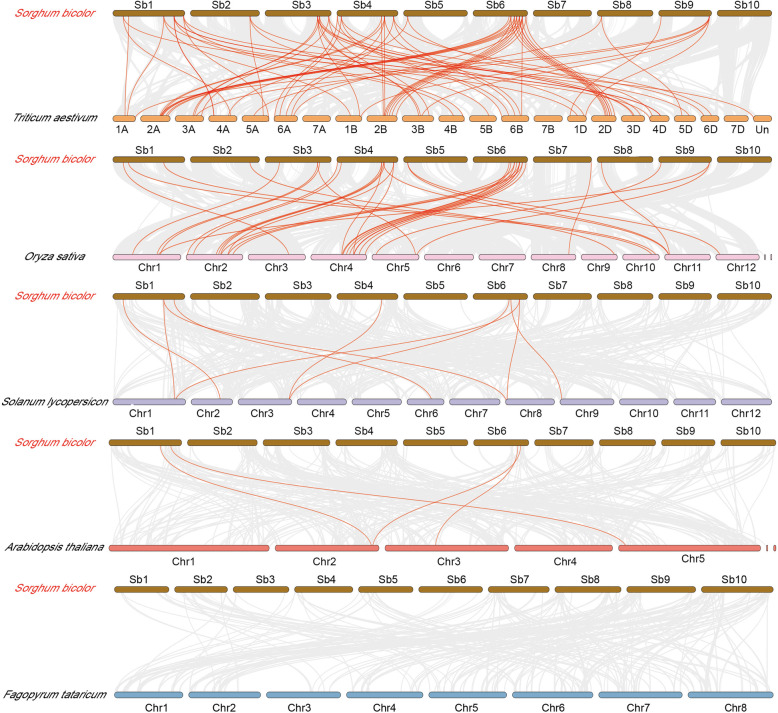
Synteny analyses of the *trihelix* genes between *S. bicolor* and five representative plant species. Gray lines on the background indicate the collinear blocks in *S. bicolor* and other plant genomes, red lines highlight the syntenic *S. bicolor trihelix* gene pairs

To examine the gene replication mechanism of sorghum trihelix family, we constructed six comparison system diagrams between sorghum and five representative species, including two dicotyledonous plants (*Arabidopsis* and tomato) and three monocotyledonous plants (buckwheat, wheat, and rice) (Fig. 4). From the details provided in Additional File 4 (Table S4), the number of collinear genes between sorghum and wheat, rice, tomato, and *Arabidopsis* were 27, 26, 5, and 4, forming 73, 35, 9, and 4 homologous gene pairs, respectively. By comparing the diagrams, we found that sorghum was the most similar with wheat and the least similar with buckwheat, which might be closely associated with the phylogenetic evolutionary relationship among them. *SbTH03* gene showed collinearity in two monocots and two dicots, indicating that *SbTH03* may be conserved in gene expansion induced by monocotyledon and dicotyledon differentiation, and play an important role in plant evolution and environmental adaptation. A total of 16 *SbTH* genes (*SbTH01/12/13/15/ 17/20/22/23/24/27/28/30/32/33/36/39*) were unique to monocots, indicating that these genes might have evolved after differentiation of monocots. Some *SbTH* genes were found to be associated with five synonymous gene pairs, including *SbTH15/32/40*. These genes may play a key role in the *trihelix* gene family during evolution. To better understand the evolutionary role of *SbTH* gene family, we performed Tajima D neutrality test (Additional File 5: Table S5). The results showed that the Tajima D value was far from 0, indicating that this gene family was strongly selected in the evolution of sorghum.

### Expression patterns of *SbTH* genes in different tissues and organs

**Fig. 5:**
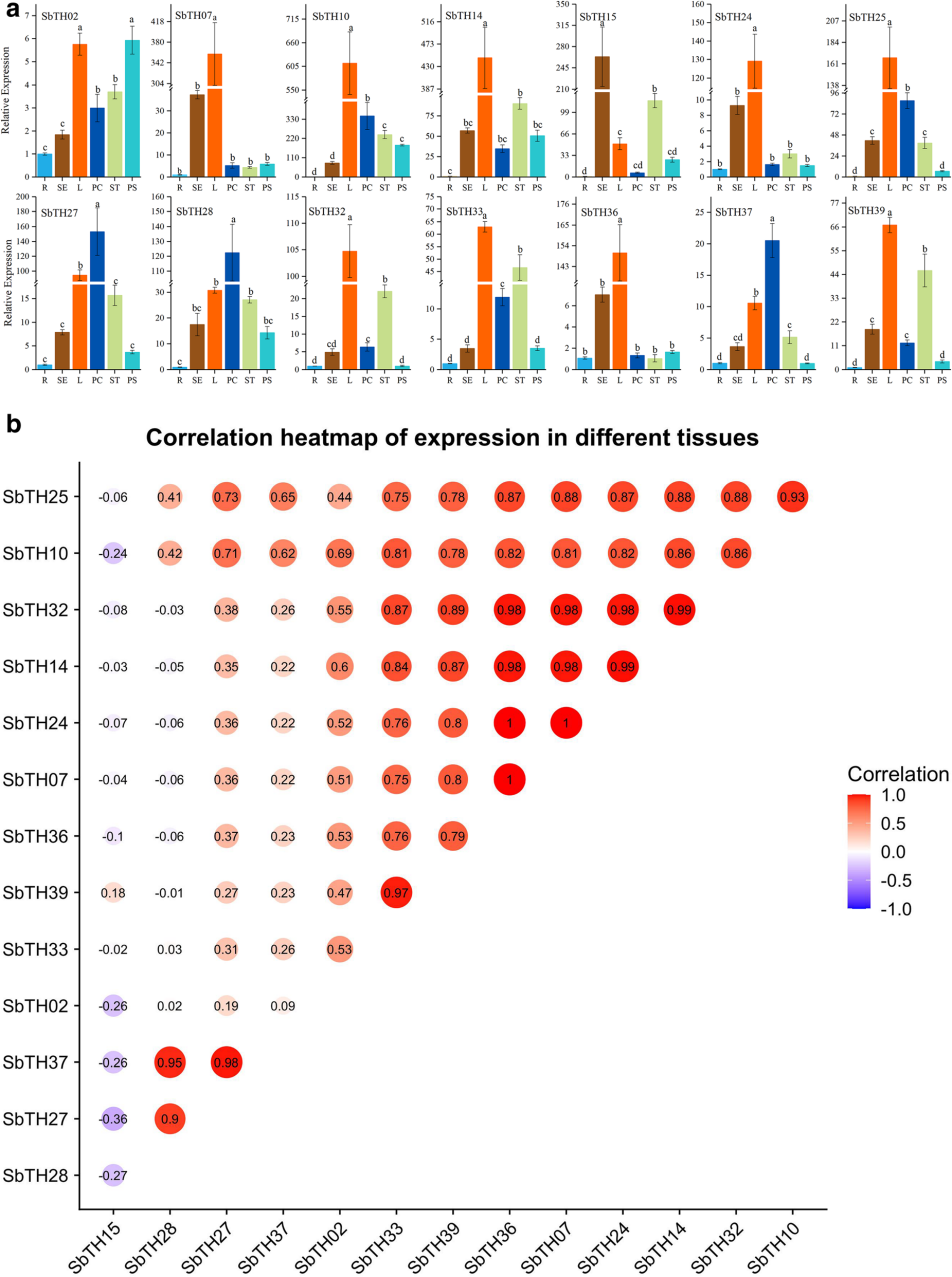
Tissue-specific gene expression and correlation between gene expression patterns of 14 sorghum *trihelix* genes **a** The expression patterns of 14 sorghum *trihelix* genes in the root (R), stem (SE), leaf (L), pericarp (PC), stamen (ST), and pistil (PS) tissues were examined by qPCR. Error bars were obtained from three measurements. Lowercase letter(s) above the bars indicate significant differences (α = 0.05, LSD) among the treatments. **b** The red round spot: positively correlated, the purple round spot: negatively correlated. The deepest and largest red round spot indicate a significant correlation at the 0.05 level.

In the plants that have been studied and reported, the functional studies of many genes indicate that *trihelix* genes play a key role in crop growth and development [34]. To understand the physiological role of *SbTH* genes in sorghum growth and development, the expression levels of two selected genes from the seven subfamilies in different sorghum organs and tissues was examined using quantitative reverse transcription polymerase chain reaction (qPCR). The expression profiles of the *SbTH* genes in selected tissues, including of root, stem, leaf, pericarp, stamen, and pistil, are shown with histograms (Fig. 5a). The *SbTH* genes were highly expressed in specific tissues and organs, indicating that SbTH family members had multiple functions in the growth and developmental stages of sorghum. We observed that 10 *SbTH* genes (*SbTH02/07/10/14/24/25/32/33/36/39*) were relatively highly expressed in sorghum leaves, five (*SbTH10/25/27/28/37*) were relatively highly expressed in sorghum pericarps, and three (*SbTH10/14/15)* were relatively highly expressed in the stems,stamens and pistils. The relative expression of *SbTH10* was the highest in leaves, pericarps, stamens and pistils, and *SbTH15* was the highest in stems. Generally, the relative expression of the 14 *SbTH* genes in the sorghum seedling roots was low (Fig. 5a).

Furthermore, we examined the correlation between the expression profiles of the 14 *SbTH* genes, and the result showed that majority of the *SbTH* genes were positively related, especially these *SbTH* genes (*SbTH25*/*10*/*32*/*14*/*24*/*07/36*/*39*) that were significantly correlated with several other *SbTH* genes. *SbTH15* was negatively correlated with 12 *SbTH* genes except *SbTH39*, while *SbTH28* was negatively correlated with *SbTH32/14*/*24*/*36*/*39*. Additionally, *SbTH28*, *SbTH27*, and *SbTH30* were significantly positively correlated with one another. The correlation coefficient of *SbTH24*, *SbTH07*, and *SbTH36* was 1 (Fig. 5b).

### Expression patterns of *SbTH* genes in response to abiotic stress

**Fig. 6:**
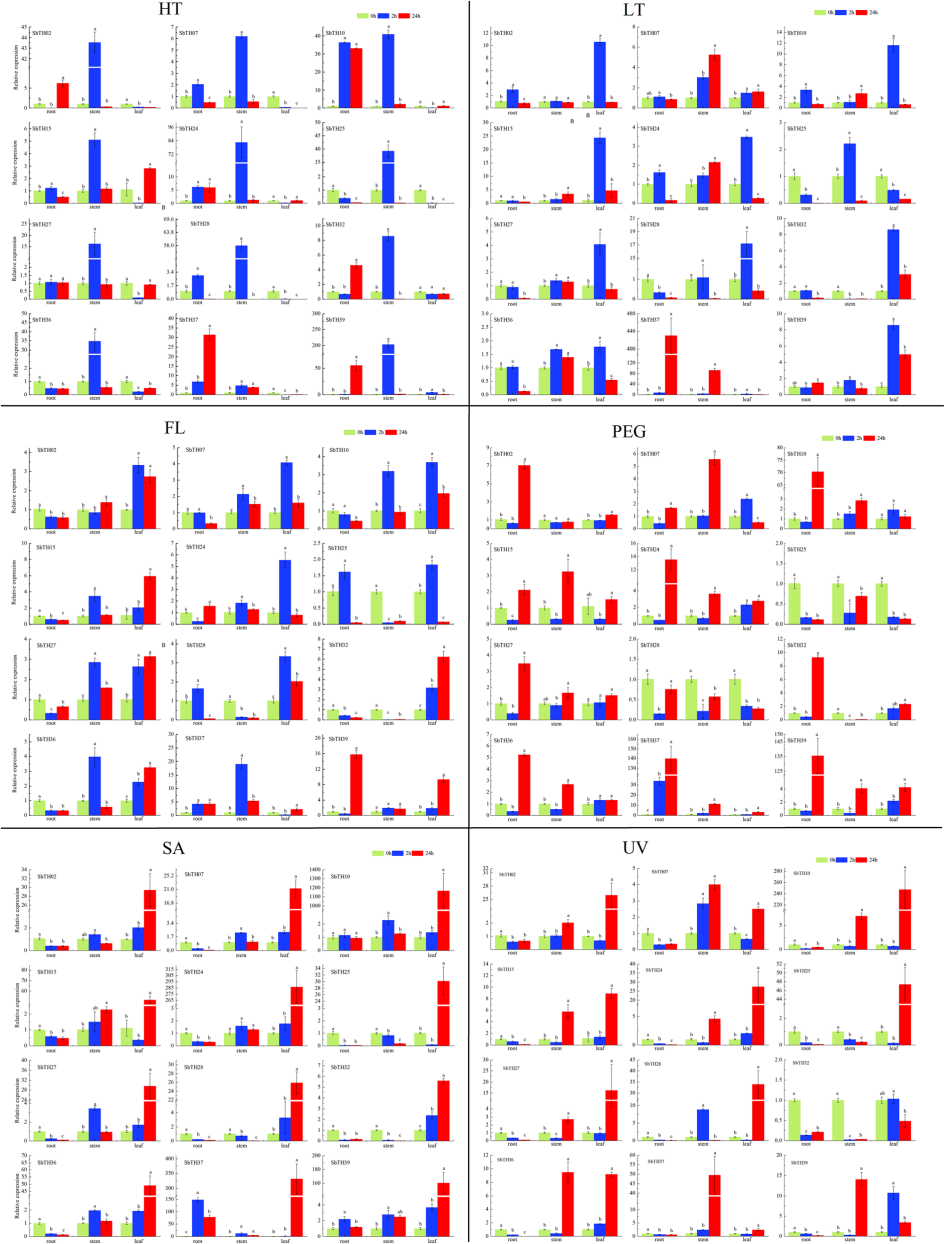
Correlation coefficient diagram of relative expression of 12 *thrihelix* genes in roots, stems and leaves of sorghum seedlings treated with 2 h (a) and 24 h (b) under different abiotic stress (FL: Flooding, HT: High temperature, LT: Low temperature, PEG: Osmotic, SA: Salt, UV: Ultraviolet radiation). The red round spot: positively correlated, the blue round spot: negatively correlated. The deepest and largest red round spot indicate a significant correlation at the 0.05 level

**Fig. 7:**
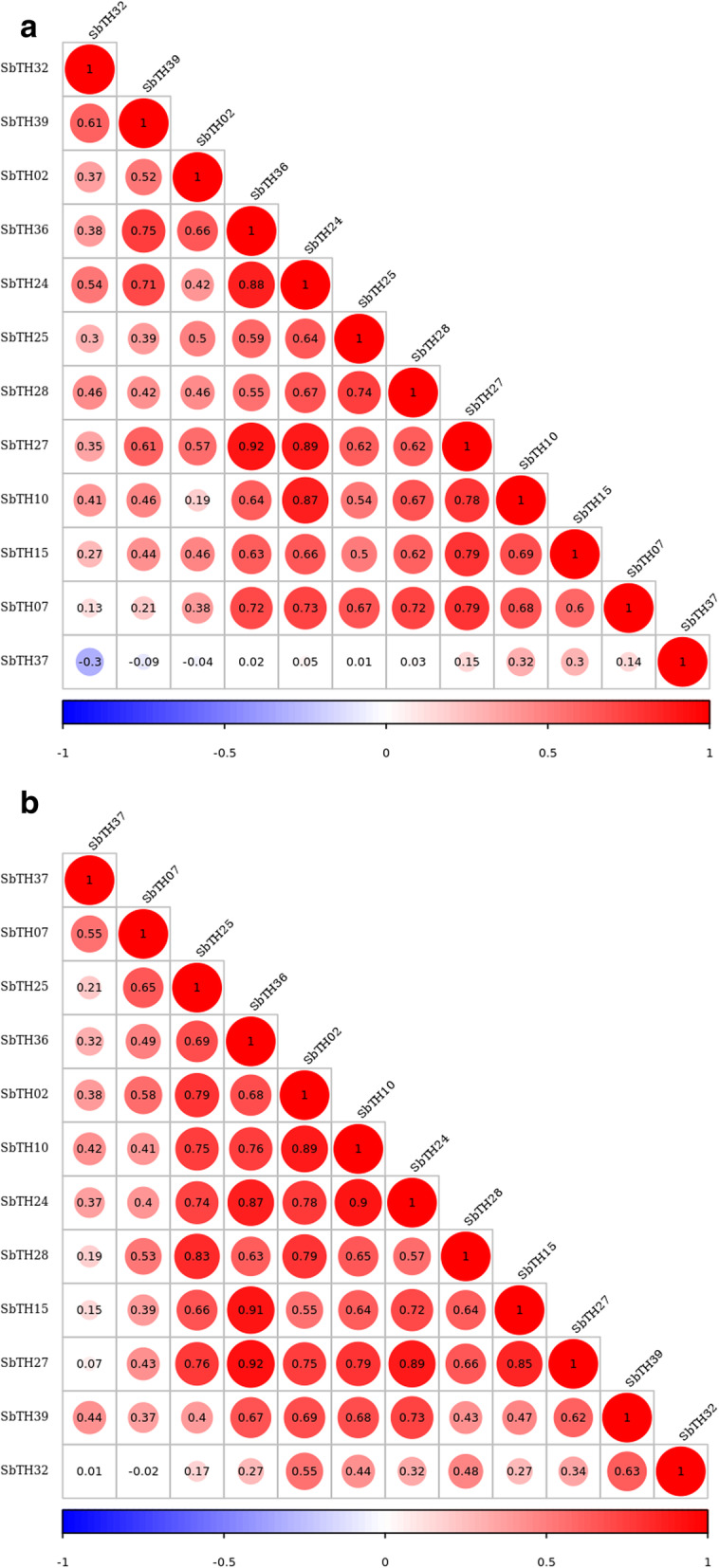
Expression patterns of 12 *S. bicolor trihelix* genes in roots, stems and leaves of sorghum seedlings treated with 2 h and 24 h under different abiotic stress (FL: Flooding, HT: High temperature, LT: Low temperature, PEG: Osmotic, SA: Salt, UV: Ultraviolet radiation) were examined by qPCR. Error bars were obtained from three measurements. Lowercase letter above the bar indicates significant difference (α = 0.05, LSD) among the treatments

To determine the role of *SbTH* genes in abiotic stress responses, we examined the expression profiles of 12 representative genes from the seven subfamilies under different abiotic stress conditions (high temperature, low temperature, osmotic, flooding, salt, and ultraviolet radiation) using qPCR. Figure 6 shows that most of the *SbTH* genes were expressed in different organs of sorghum after 2 h under high temperature, low temperature, and water flooding, whereas most of the *SbTH* genes were expressed after 24 h under osmotic, salt, and ultraviolet irradiation. There was a significant upregulation of the expression profiles of the 12 *SbTH* genes in the stems after 2 h under high temperature stress, however, *SbTH37* expression was significantly upregulated in the roots after 24 h under high temperature stress. Most of the *SbTH* genes were highly expressed in the leaves after 2 h exposure to low temperature, while *SbTH07* and *SbTH37* were highly expressed in the stems and roots, respectively, after 24 h exposure to low temperature. Furthermore, most of the genes were upregulated in the roots after 24 h exposure to osmotic condition, however, *SbTH25* and *SbTH28* were relatively downregulated. Most of the genes (*SbTH02*/*07*/*10*/*24*/*25*/*28*) were upregulated in the leaves after 2 h exposure to flooding, while *SbTH15*, *SbTH27*, and *SbTH32* were upregulated after 24 h exposure. Additionally, *SbTH36* and *SbTH37* were upregulated in the stems after 2 h exposure to flooding, while *SbTH39* was upregulated in the roots after 24 h of exposure. Most of the genes (*SbTH02*/*10*/*15*/*24*/*25*/*27*/*28*) were upregulated in the leaves, while *SbTH07*, *SbTH37*, and *SbTH39* were upregulated in the stems after 24 h exposure to ultraviolet radiation. However, *SbTH32* was not upregulated in the roots, stems, and leaves. All the *SbTH* genes were highly expressed in leaves after 24 h of exposure to salt stress, in which the relative expression of *SbTH10/24/37* reached hundreds or even thousands fold. Figure 7 shows the correlation coefficient diagram of 12 *thrihelix* genes transcriptional expression fold changes in response to abiotic stress. According to the analysis, there was a significant correlation between the relative expression of most genes at 2 h and 24 h, but no significant correlation between *SbTH32 /37* and other genes. After 2 h of treatment, there was a negative correlation between *SbTH32* and *SbTH37*, no significant correlation between *SbTH37* and the other 11 *thrihelix* genes, between *SbTH32* and *SbTH39/24* was significant (Fig. 7a). After 24 h of treatment, the relative expression of *SbTH37/07/32* was not significant compared with most other genes, between *SbTH37* and *SbTH07* was significant, between *SbTH32* and *SbTH37/07/25/36/10/24/15/27* was not significant, between *SbTH07* and *SbTH10/24/15/27/39/21* was not significant (Fig. 7b).

## Discussion

Sorghum is the dietary staple of over 500 million people in more than 30 countries in the tropics and semitropics [35]. Sorghum is a typical C4 crop and an important raw material for livestock nutrition and brewing industry. The sorghum reference genome was published in 2009 [36], however, whole genome studies of sorghum *trihelix* gene family have not been published. In the present study, 40 *SbTH* genes were identified in sorghum, similar to the number of *SbTH* genes in tomato and rice [12, 24]. Trihelix family genes were previously classified into three distinctive subfamilies (GTα, GTβ, and GTγ) [37]. Kaplan-Levy et al. classified *trihelix* genes from rice (*Oryza sativa*) and *Arabidopsis* into five clades namely GT-1, GT-2, SH4, SIP1, and GTγ [33]. Recently, a new subfamily, GTδ, was identified in tomato (*Solanum lycopersicum*) and rice [12, 24]. In the present study, phylogenetic analysis showed that sorghum *trihelix* genes were classified into six subfamilies (GT-1, GT-2, SH4, SIP1, GTγ, GTSb8) (Fig. 1). Based on the constructed phylogenetic tree, we identified at least one trihelix protein from *S. bicolor* in each subgroup of AtTHs and OsTHs [20, 24], indicating that the time of differentiation of the trihelix family may have been earlier than differentiation of monocotyledons and dicotyledons. The *trihelix* genes within the reported subfamilies may play a fundamental role in the tissue development, environmental adaptation and gene evolution in dissimilar plant species, including *Arabidopsis thaliana* [20], tartary buckwheat [30], *Brachypodium distachyon* [38], Moso bamboo [39], and wheat [29]. Compared to *A. thaliana*, the group SbSH4 (6, 15.0%) has more members, and indicates that those SbSH4 members may have undergone stronger partial differentiation in the long-term evolutionary process. A new subfamily (GTSb8) and three new ‘orphan genes’ were found in sorghum, suggesting the possibility of further differentiation of TH family in sorghum. This new cluster of ‘GTSb8’ indicates the complexity of genetic structure and physiological function of *trihelix* gene members. However, more evidence is needed to determine whether the new cluster is unique to C4 plants.

The results of motif composition and gene structure analysis of the *trihelix* genes were consistent with the phylogenetic classification results. The *SbTH* genes in the GTSb8 and ‘*orphan* gene’ subfamilies had only the trihelix domain (GT domain), whereas all members of the SH4, SIP1, and GTγ subfamilies had MYB DNA-binding domains. The similarity of most members in the same subfamily indicate that the conserved motifs may play a critical role in the functions of specific groups. Sequence distribution indicated that genes with the same motif may be generated by gene amplification in the identical population, which is similar to the report in chrysanthemum [40]. Among the seven subfamilies, GT-1 and GT-2 have been examined in previous studies, and their homology is much higher than that of other subfamilies [41]. Gene duplication is one of the major evolutionary mechanisms for generating novel genes that help organisms adapt to different environments [42, 43]. Generally, gene families expand mainly by tandem and segmental duplications [42]. Tandem and segmental duplications are key factors in enriching protein function and promoting gene evolution and expansion [44]. Sorghum had fewer *trihelix* genes than did soybean (71), *Populus trichocarpa* (56), and *Brassica napus* (52) [45–47], but more *trihelix* genes than did chrysanthemum (20), buckwheat (31), and tomato (36) [12, 27, 30]. This difference may be due to whole-genome duplication event that occurred after the earliest ancestors of the other species diverged. It is assumed that the occurrence and evolution of some *SbTH* genes may be driven by these fragment duplication events, which is similar to the report on *Populus trichocarpa* [46]. Based on the results of chromosome distribution, there was no *SbTH* gene on chromosome 10, indicating that the *SbTH* gene family may have been affected by gene deletion during the evolutionary process [48]. A similar phenomenon was reported in the rice and soybean *trihelix* gene families, which contained only 6 and 13 pairs of duplicated genes among a total of 41 (29.3%) rice *trihelix* genes and 71 (36.6%) soybean *trihelix* genes, respectively [21, 24]. Some *SbTH* gene deletions can be attributed to dynamic changes after fragment duplication, which is consistent with the findings of *Populus trichocarpa* [46]. In this study, tandem repeat events (*SbTH04 / SbTH05*) contributed less to the increase of sorghum trihelix membership than segmental duplicated (12 *trihelix* genes, 30.0%). Further analysis of these Trihelix members revealed that they were all linked within subfamilies. Therefore, some Trihelix genes may be generated by some replication events, which further confirms that replication events may be an important mechanism for the rapid expansion of Trihelix family members in plants.

In addition, we analyzed the exon and intron structures of 40 identified *SbTH* genes (Fig. 2, Attached File 1: Table S1). The number of exons in each gene ranged from 1 to 17 (Fig. 2A/2B). The proportion of *SbTH* gene without introns (15, 37.5%) was close to that of rice [24]. Interestingly, most of the intron-free genes are distributed in the GTγ subfamily and the SIP1 subfamily, which are similar to *Arabidopsis thaliana* [20]. A certain number of introns can increase the length of genes and the frequency of recombination between genes, which is beneficial to the evolution of species. However, intron-free genes tend to respond quickly to changes in the environment [49]. The lowest average number of exons was observed in the GTγ subfamily, whereas the highest was observed in the GT1 subfamily, which is consistent with the result in wheat [29] and buckwheat [30]. As the largest subfamily, the motif compositions of the SIP1 subfamily (most of the members of this clade shared motifs 1, 2, 5, 7, and 8) were different from that of other subfamily members, whose motif compositions were similar to that in cabbage, chrysanthemum [27], wheat [29], and *Medicago truncatula* [50]. The SIP1 subfamily members may have more complex and diverse functions than other subfamily members in sorghum.

Previous studies have shown that trihelix TF family is widely involved in the development of plant organs [30]. The expression levels of *trihelix* gene in sorghum stem, root, leaf and flower were determined by qPCR. As shown in Fig. 5a, most of trihelix gene members showed significant differential expression (more than 2-fold difference). *SbTH02*, is classified into subfamily GT2, has the highest expression levels in leaves and pistils, which is similar to the expression pattern of homologous gene *AT5G03680.1*, which regulates collective leaf structure and inflorescence development in *Arabidopsis* [49, 51]. As expected, the subfamily GT1 members, *SbTH32* and *SbTH39*, are highly expressed in leaves and stamens, which was consistent with the expression pattern of the homologous gene *AT1G13450* [52]. In addition, the expression of *SbTH07*, *SbTH10*, *SbTH14*, SbTH25, *SbTH33*, and *SbTH36* in leaves of sorghum were significantly higher than those in roots, stems and pericarps. These tissue-specific *trihelix* genes may play a role in the growth and differentiation of corresponding organs, but more experiments are needed to verify the function of these genes [53]. In addition, some *SbTH* genes showed significant positive correlation, such as *SbTH27* and *SbTH28* (Fig. 5b). For example, *SbTH27*, *SbTH28* and *AtTH13*, both belonging to subgroup SIP1 and having similar motif components (Fig. 2). The expression of *SbTH27* and *SbTH28* in pericarp of millet were significantly higher than those in roots, stems and leaves, and their expression pattern is similar to that of *AtTH13* [54]. Therefore, we can further verify the possible relationship between these genes and pericarp development through some experiments. The expression levels of some SbTH members were significantly positively correlated, indicating that they may play a synergistic effect in six sorghum organs (Fig. 5b).

To further explore the physiological role of the trihelix family in environmental adaptation, we systematically analyzed the expression of 12 SbTHs in sorghum seedlings under six stresses (Fig. 6). For example, under NaCl stress, the expression levels of 10 *SbTH* genes were significantly up-regulated in roots, 8 in stems, and 7 in leaves and which may help sorghum adapt to drought conditions. In this study, under UV, PEG and NaCl treatment, *SbTH15* showed obvious induction effect at seedling stage, and its expression level in stems and leaves were significantly increased. *AtTH26* and *SbTH15*, are the members of subfamily GT2, have similar motif composition. Previous studies have shown that *AtTH26* (*At5G28300*) can be induced by NaCl, drought, cold, and abscisic acid, and highly expressed in Arabidopsis inflorescence and leaves to help improve its resistance to adversity [55]. *ShCIGT*, a cold-inducible gene isolated from wild tomato, contributes to the improvement of abiotic stress tolerance in tomato [56]. Similarly, the expression of *SbTH39* was significantly up-regulated in almost all abiotic stresses, which may enhance the adaptability of sorghum to the environment in a similar pattern. In *Arabidopsis*, the GT1 cis element interacts with the GT-1-like transcription factor *AtGT-3b* in vitro and in the yeast system. Transcription of *AtGT-3b* was also rapidly induced within 30 min after sodium chloride treatment, thus helping to enhance its resistance to salt stress [57]. Yoo et al. [58] found that *GT2-like 1* (*GTL1*) in *Arabidopsis thaliana* is a transcriptional suppressor for promoter of STOMATAL DENSITY AND DISTRIBUTION 1 (*SDD1*), which can negatively regulate stomatal development and transpiration [57, 59]. Interestingly, *SbTH37* is highly expressed in response to almost all stresses in some tissues, suggesting that some new evolutionary directions in sorghum may be the result of multiple adaptations to the environment. In addition, many studies have shown that TH-TFs are not only involved in response to abiotic stress, but also in disease resistance [15]. After being infected by Magnaporthe grisea, the *GT-1-like* gene in rice, *rml1*, can be rapidly up-regulated in seedlings to reduce the damage of the pathogen [14]. The *GTL1* gene plays a key role in the MPK4 pathway in *Arabidopsis* by regulating the balance of salicylic acid and acting as a bacteria-induced immune factor [60]. *SbTH28*, a member of the subfamily GTSb8, was significantly down-regulated in roots under six stresses. This shows that it may actively participate in the response to abiotic stress. In summary, the expression patterns of SbTH members of the six subfamilies show great differences, which indicates that different genes may play a role with unique physiological functions. These results indicate that the *trihelix* gene family may play an important role in the tissue development and abiotic stress of sorghum, which needs further experimental verification.

## Conclusion

In summary, the study is the first genome-wide analysis of *trihelix* genes in sorghum. We identified 40 *trihelix* genes in sorghum, which were classified into seven subfamilies and distributed in nine chromosomes. Additionally, we identified one pair of tandem duplication gene and seven pairs of segmental duplication genes in the *SbTH* gene family, indicating that gene duplication is the main force driving *trihelix* gene evolution in sorghum. Based on the expression profiles of the *SbTH* genes in different organs and tissues of sorghum under different abiotic stress conditions, some of the key candidate genes were screened out. For example, *SbTH10*, *SbTH37*, and *SbTH39* may play important roles in the tissues development and abiotic stresses of sorghum. The findings of our study serve as a basis for further investigation of the functions of *SbTH* genes and provide candidate genes for increasing sorghum yield.

## Methods

### Gene identification

We downloaded the complete *S. bicolor* genome sequence (Accession: GCA_000003195) from the Ensembl Genomes website (http://plants.ensembl.org/Sorghum_bicolor/Info/Index). The trihelix family members were identified by two BLASTp searches [61, 62]. First, all possible trihelix proteins with score value ≥100 and e-value ≤1^− 10^ were identified from the *S. bicolor* genome, referring to trihelix protein sequences of *A. thaliana* by BLASTp search. Second, the HMM profile consistent with the trihelix domain was obtained from the Pfam protein family database (http://www.pfam.sanger.ac.uk). Candidate SbTH proteins containing the trihelix were screened out using HMMER3.0 (default parameters) with a cutoff of 0.01 (www.plants.ensembl.org/hmmer/index.html) [63] and SMART http://www.smart.embl-heidelberg.de). [64, 65]. In addition, information on basic features of the trihelix proteins of the *SbTH* gene family, including coding sequence length, isoelectric point, protein molecular mass, and subcellular localization, was obtained from the ExPasy website (http://web.expasy.org/protparam/).

### *Trihelix* gene structure

The trihelix domain sequences of the characterised SbTH proteins were used to create multiple protein sequence alignments using ClustalW with default parameters [66]. The deduced amino acid sequences in the trihelix domains were then adjusted manually using Mega 6.0 and GeneDoc 2.7. The exon/intron structures of the *SbTH* genes were generated by the Gene Structure Display Server (GSDS: http://GSDS.cbi.pku.edu.cn) [67]. To compare the differences in SbTH proteins, the conserved motifs of the trihelix proteins were determined. The analysis of the conserved protein motifs in SbTH proteins was performed with the protein conserved motif online search program MEME (http://meme-suite.org/tools/meme) [68, 69]. The optimization parameters were set to the maximum number of motifs of 10 and the motif breadth as 6 to 200 amino acid residues [62, 69, 70].

### Chromosomal distribution and gene duplication

All *SbTH* genes were mapped to *S. bicolor* chromosomes based on physical location information from the database of the *S. bicolor* genome using Circos [71]. The detection and study of the gene duplication events in *SbTH* genes were performed using the multiple collinear scanning toolkits (MCScanX) with default parameters [72]. We analysed the homology of the *trihelix* genes between *S. bicolor* and five plants (*A. thaliana, V. vinifera, S. lycopersicum, B. distachyon, O. sativa subsp. indica,* and *Z. mays*) using Dual Synteny Plotter (https://github.com/CJ-Chen/TBtools). Non-synonymous (ka) and synonymous (ks) substitutions of each duplicated *trihelix* gene were calculated using Ka/Ks-Calculator 2.0 [73].

### Phylogenetic analysis and classification of *trihelix* gene family

The *Arabidopsis* trihelix and SbTH protein sequences were used for multiple amino acid sequence alignments using MEGA X software, and we used the NJ method with a bootstrap value of 1000 replicates and default parameters to construct the unrooted phylogenetic tree. The full-length amino acid sequences of the trihelix proteins (Additional file 1: Table S1) of SbTH in *A. thaliana, V. vinifera, S. lycopersicum, B. distachyon, O. sativa subsp. indica,* and *Z. mays* were used to construct the phylogenetic trees. The trihelix protein sequences were obtained from the UniProt database (UniProthttps://www.uniprot.org/). The identified *SbTH* genes were classified into different subfamilies.

### Plant materials, growth conditions, and abiotic stress in *S. bicolor*

*Sorghum bicolor* ‘Hongyingzi’ was used for this study. The sorghum plants have been under cultivation in the greenhouse of Guizhou University since 2019. The plants were grown in pots filled with soil and vermiculite (1:1) in a growth chamber under a 16 h/25 °C day and 8 h/20 °C night regime and 75% relative humidity. We collected the stems, roots, leaves, pericarp, stamen, and pistil from five healthy sorghum plants. The organs were quickly fixed in liquid nitrogen and stored at − 80 °C until further analysis. The expression profiles of selected *SbTH* genes in different organs of 21-d-old sorghum plants after 2 h and 24 h under different abiotic stress conditions were examined using qPCR analysis. The seedlings were subjected to salt (900 mM NaCl), water flooding (whole plant), osmotic (30% PEG6000) [74, 75] [1], UV exposure (70 μW/cm^2^, 220 V, 30 W), high temperature and low temperature stress conditions (The plants were placed in light incubators at 40 °C and 4 °C, with 80% light, 16 h during the day, 8 h at night and 75% humidity.). Each stress treatment was performed with five replicates. Sorghum plants used for later sampling were planted in the teaching experimental field of Guizhou University, and the cultivation and management measures were consistent with the field production.

### Total RNA extraction, cDNA reverse transcription, and qPCR analysis

Total RNA of each sample was extracted using a plant RNA extraction kit (TIANGEN DP441), and the sequences were used for cDNA library construction. qPCR SYBR Green Premix (Vazyme, China) was used to conduct qPCR analysis in a CFX™ real-time PCR detection system (Bio-Rad, USA). The primer sequences used were designed by Primer 5.0 (Additional File 6: Table S6). We used the *Actin* gene, which was stably expressed at each growth stage in almost all tissues, as the internal control [76]. The *ACTIN* gene was used as calibration to detect three technical repeats of the three biological repeats, and 2^-ΔΔCT^ method was used to analyze the expression [77].

### Statistical analysis

Data obtained during the study were subjected to analysis of variance (ANOVA) using SPSS software (IBM Corporation). Mean values were compared using Fisher’s least significant difference (LSD) test at 0.05 significance level. The histograms were drawn using Origin 8.0 software (OriginLab Corporation, Northampton, Massachusetts, USA).

## Supplementary Information

**Additional file 1 Table S1.** List of the 40 *SbTH* genes identified in this study. (XLS 143 kb)

**Additional file 2 Table S2.** Analysis and distribution of the conserved motifs in *S. bicolor* trihelix proteins. (XLS 22 kb)

**Additional file 3 Table S3.** Seven pairs of segmental duplicated *S. bicolor trihelix* genes. (XLS 22 kb)

**Additional file 4 Table S4.** One-to-one orthologous gene relationships between *S. bicolor* and other plants. (XLS 43 kb)

**Additional file 5 Table S5.** Results of Tajima’s D neutrality test.

**Additional file 6 Table S6.** Primer sequences for qPCR.

**Additional file 7 Fig. S1.** Schematic representation of the chromosomal distribution of *S. bicolor trihelix* genes. Vertical bars represent the chromosomes of *S. bicolor*. The chromosome number is indicated to the left of each chromosome. The scale on the left represents chromosome length.

**Additional file 8 Fig. S2.** Phylogenetic relationship and motif composition of the trihelix proteins of *S. bicolor* and five different plant species. Outer panel: An unrooted phylogenetic tree constructed using Geneious R11 with the NJ method. Inner panel: Distribution of the conserved motifs in trihelix proteins. The differently coloured boxes represent different motifs and their positions in each trihelix protein sequence. The sequence information for each motif is provided in Additional File 2: Table S2.

## Data Availability

The entire *Sorghum bicolor* genome sequence (Accession:GCA_000003195) information was obtained from the Ensembl Genomes website (http://ensemblgenomes.org/). The *Sorghum bicolor* materials (Hongyingzi) used in the experiment were supplied by Prof. Mingjian Ren of Guizhou University. This variety was approved in the 5th Crop Variety Approval Committee of Guizhou Province on June 19, 2008, the second chairperson meeting, and was stored in the grain crop germplasm bank of College of Agriculture, Guizhou University. It was numbered GZ234. The permission of this material has been approved by the Guizhou University. The datasets supporting the conclusions of this article are included in the article and its Additional files.

## Abbreviations

TH: Trihelix
SbTH: *Sorghum bicolor* Trihelix
GAPDH: Glyceraldehyde-3-phosphate dehydrogenase
qPCR: Quantitative real-time polymerase chain reaction
TF: Transcription factor
AtTH: *Arabidopsis thaliana* Trihelix
HMM: Hidden Markov Model
PI: Isoelectric point
LG: Linkage group
Mw: Molecular weight
PSL: Predicted Subcellular Location
N: Nucleus
Ch: Chloroplast
Ec: Extracellular
Cy: Cytoplasm
PL: Protein length
