# Supplementary figures and images for: Genome-wide identification and expression profile analysis of trihelix transcription factor family genes in response to abiotic stress in sorghum [Sorghum bicolor (L.) Moench]
Source: BMC Genomics. 2021 Oct 14;22:738. doi: 10.1186/s12864-021-08000-7 (PMC8515681; doi:10.1186/s12864-021-08000-7)

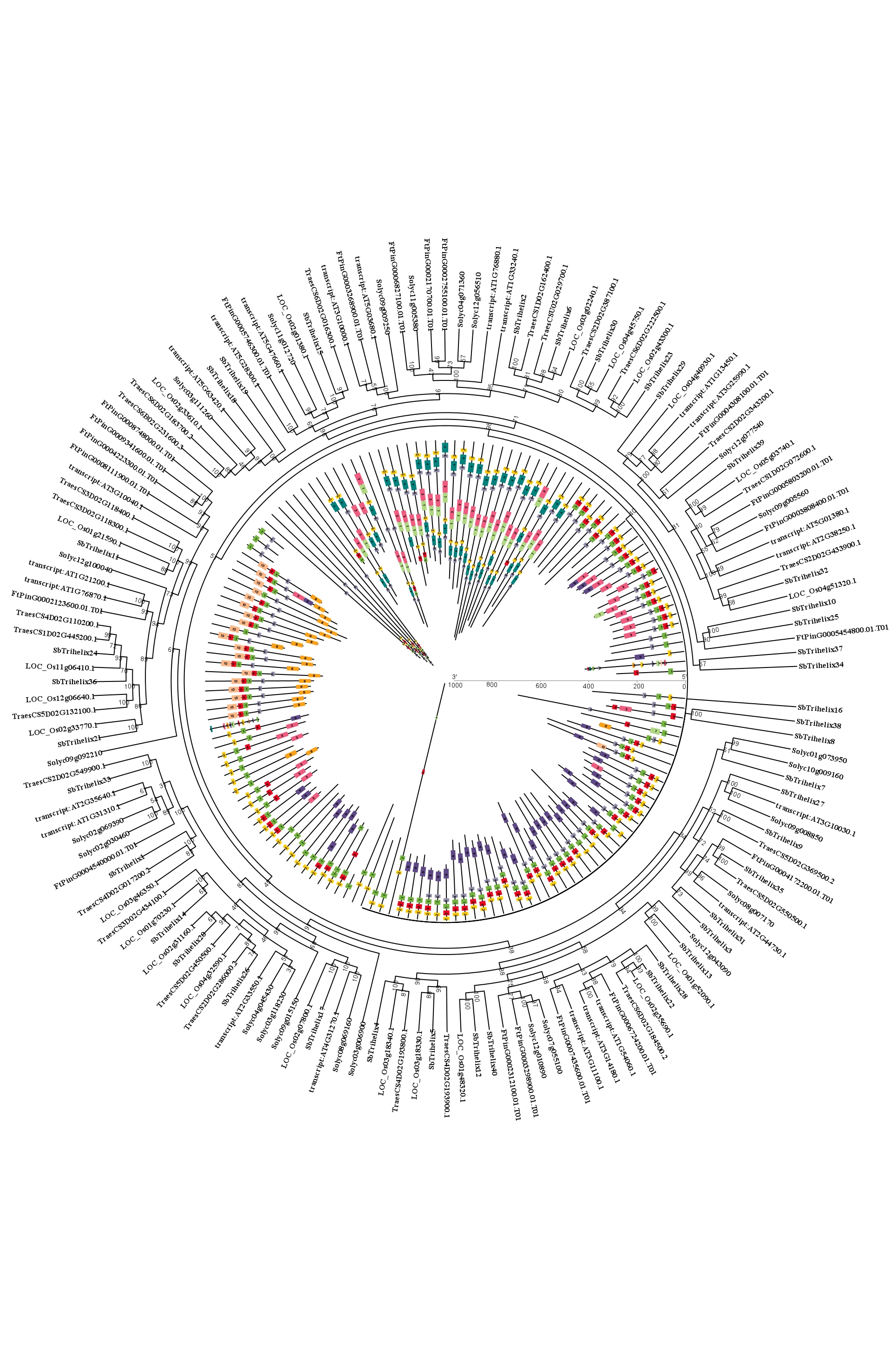

Supplement: Supplementary file 8 — Additional file 8 Fig. S2. Phylogenetic relationship and motif composition of the trihelix proteins of S. bicolor and five different plant species. Outer panel: An unrooted phylogenetic tree constructed using Geneious R11 with the NJ method. Inner panel: Distribution of the conserved motifs in trihelix proteins. The differently coloured boxes represent different motifs and their positions in each trihelix protein sequence. The sequence information for each motif is provided in Additional File 2: Table S2. [file 12864_2021_8000_MOESM8_ESM.jpg]
